# Study on the synthesis of novel 5-substituted 2-[2-(pyridyl)ethenyl]-1,3,4-oxadiazoles and their acid–base interactions

**DOI:** 10.1007/s00706-014-1355-x

**Published:** 2014-12-20

**Authors:** Agnieszka Kudelko, Karolina Jasiak, Krzysztof Ejsmont

**Affiliations:** 1 Department of Chemical Organic Technology and Petrochemistry, The Silesian University of Technology, Krzywoustego 4, 44100 Gliwice, Poland; 2 Faculty of Chemistry, Opole University, Oleska 48, 45052 Opole, Poland

**Keywords:** Heterocycles, Basicity, Cyclizations, 1,3,4-Oxadiazoles, 3-(Pyridyl)acrylohydrazides, p*K*_A_ Ionization constants

## Abstract

**Abstract:**

A series of novel 5-substituted 2-[2-(pyridyl)ethenyl]-1,3,4-oxadiazoles were efficiently synthesized by cyclocondensation of the appropriate 3-(pyridyl)acrylohydrazides with triethyl orthoesters in the presence of glacial acetic acid. The products were identified by means of spectroscopic methods and their p*K*_A_ ionization constants were determined. The influence of substituents on the basicity of the pyridine system has been discussed.

**Graphical Abstract:**

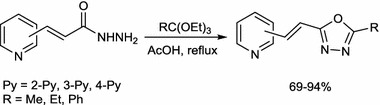
 
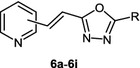

## Introduction

1,3,4-Oxadiazoles belong to the group of five-membered aromatic heterocycles, containing one oxygen and two nitrogen atoms. Many of these compounds exhibit a wide range of pharmaceutical and biological activities such as antibacterial, antiviral, anti-inflammatory, analgesic, or anticonvulsant [1–6]. Additionally, 1,3,4-oxadiazole derivatives act as potential agents in the treatment of cancer and AIDS [7–10]. They are also used extensively in agriculture as herbicides, fungicides, or insecticides [11, 12]. These heterocyclic molecules are applied in the production of heat-resistant polymers, blowing agents, optical brighteners, and anti-corrosion agents [13–16]. Conjugated *π*-electronic arrangements based on the electron-deficient 1,3,4-oxadiazole ring feature excellent electron-transporting properties with much higher quantum efficiency in comparison to conventional fluorescent emitters using silicon and its solid solutions (doped silicon). Therefore, they are used as monomers in the production of fluorescent emitters for organic light-emitting diodes, photovoltaic cells, scintillators, and photosensitive materials [13–16]. However, many of the previously investigated compounds applied in organic electronics suffer from their poor processability and low thermal and chemical stability. Due to these facts, the study on designing and synthesis of new organic conjugated materials whose physicochemical properties may be easily modified seems to be reasonable.

Synthesis of 1,3,4-oxadiazoles has been first described by Ainsworth in the 50s last century [17]. The most popular methods to synthesize 1,3,4-oxadiazole scaffold involve the use of *N*,*N*′-diacylhydrazines or *N*-acylhydrazones (Scheme 1). Typically, cyclodehydration of *N*,*N*′-diacylhydrazines is carried out using reagents such as PPA [18], H_2_SO_4_ [19], SOCl_2_ [20, 21], POCl_3_ [22, 23], P_2_O_5_ [24] (CF_3_SO_2_)_2_O [25], BF_3_·OEt_2_ [26] or the Burgess reagent [27]. 1,3,4-Oxadiazole derivatives may also be prepared by oxidative cyclization of *N*-acylhydrazones with oxidizing agents such as CAN [28], KMnO_4_ [29], FeCl_3_ [30], Br_2_ [31], PbO_2_ [32], chloramine T [33], HgO/I_2_ [34], hypervalent iodine reagents [35–41]. One-pot syntheses of these compounds from acid hydrazides with carboxylic acids [42] or orthoesters [43–45] in the presence of an acidic catalyst have also been reported. Other synthetic routes involve acylation and subsequent ring opening and ring closure of starting tetrazoles [46, 47], heterocyclization of semicarbazide, thiosemicarbazide and selenosemicarbazide derivatives [48–50], as well as transformation of 1,2,4-oxadiazoles under the influence of UV radiation [51]. Recently, solid phase syntheses of arrangements based on the 1,3,4-oxadiazole fragment have also been described in the literature [52–56].

**Figure:**
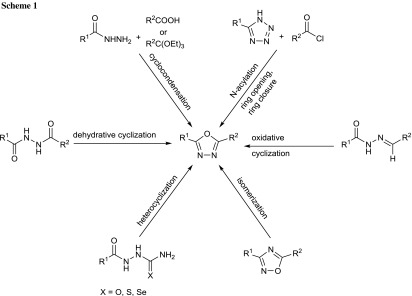

In continuation of our studies on the application of *α*,*β*-unsaturated acid hydrazides in the synthesis of conjugated 2-[2-(aryl)ethenyl]-1,3,4-oxadiazole derivatives, we investigated structures possessing the pyridylethenyl moiety at the α position [57]. Herein, we report the synthesis of three types of 3-(pyridyl)acrylohydrazides and their reactions with triethyl orthoesters. The presence of the acid-sensitive pyridyl fragment is particularly important because it allows the acid–base modification of the physical properties of the indicated structures which may serve as potential monomers for optoelectronics.

## Results and discussion

Hydrazides of selected 3-(pyridyl)acrylic acids **5a**–**5c** were used as precursors of 1,3,4-oxadiazole derivatives. These compounds were obtained from the appropriate commercially available heteroaromatic aldehydes, 2-pyridinecarboxaldehyde (**1a**), 3-pyridinecarboxaldehyde (**1b**), and 4-pyridinecarboxaldehyde (**1c**) according to a few-step procedure (Scheme 2).

**Figure:**
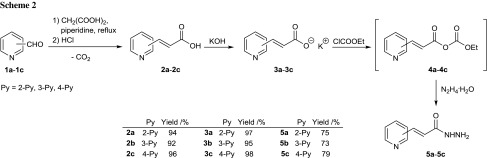

In a typical synthetic procedure, the starting aldehydes were treated with malonic acid in pyridine in the presence of piperidine as a catalyst under Knoevenagel–Doebner reaction conditions. Condensation and successive decarboxylation of intermediate dicarboxylic acids occurred giving *α*,*β*-unsaturated monocarboxylic acids, 3-(pyridyl)acrylic acids **2a**–**2c** in high yields. The resulting acids were neutralized with potassium hydroxide to form the appropriate potassium salts **3a**–**3c** which were then used in a one-pot, two-step synthesis, yielding acid hydrazides **5a**–**5c**. First, the potassium salts **3a**–**3c** were treated with ethyl chloroformate and finally excess amounts of hydrazine hydrate. The reaction conducted at low temperature in acetonitrile solution resulted in the formation of the desired hydrazides **5a**–**5c** in satisfactory yields (73–79 %, Scheme 2). The same hydrazides **5a**–**5c** were also prepared by the typical two-step transformation from the appropriate 3-(pyridyl)acrylic acids **2a**–**2c** by esterification with methanol and thionyl chloride followed by treatment with hydrazine hydrate. However, the low yields of the final hydrazides **5a**–**5c** (35–44 %) made the above synthetic procedure unattractive.

The resulting acid hydrazides **5a**–**5c** were heated with an excess of triethyl orthoesters (*R* = Me, Et, Ph; Scheme 3) in glacial acetic acid, yielding a series of 2-[2-(pyridyl)ethenyl]-1,3,4-oxadiazoles **6a**–**6i** substituted at the 5-position with a phenyl or an alkyl group that have not previously been reported in the literature. The commercially available triethyl orthoesters play the dual role of the synthon introducing the methylene carbon atom and high-boiling solvent.

**Figure:**
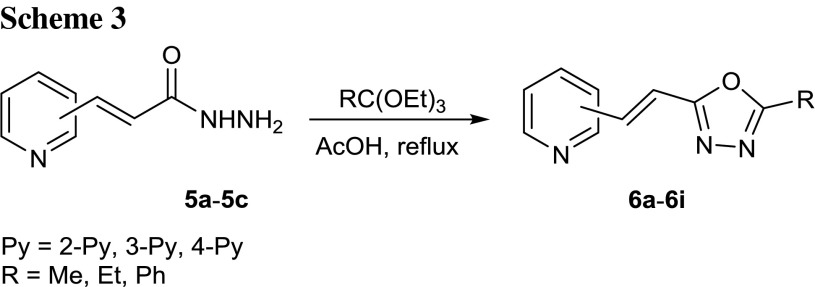

Generally, the reaction yields increased with the increasing bulk of substituent R on the orthoester. The best results were obtained in the case of derivatives with a phenyl group at the 5-position (*R* = Ph 88–94 %, Table 1), due to the presence of an extended conjugated system and a higher boiling point of triethyl orthobenzoate (b.p. 240 °C) in contrast to the boiling points of the rest of the orthoesters (b.p. 142–152 °C). The rest of 1,3,4-oxadiazoles with electron-donating alkyl groups were prepared in lower yields. We have also observed an influence of the position of the pyridine nitrogen atom on the reaction yields. The highest values were obtained in the reactions conducted with 3-(2-pyridyl)acrylohydrazide (**5a**, 74–92 %) and 3-(4-pyridyl)acrylohydrazide (**5c**, 76–94 %, Table 1).

**Table 1:** Products of the reaction of 3-(pyridyl)acrylohydrazides **5a**–**5c** with triethyl orthoesters

Product	Py	*R*	Reaction time/h	Yield^a^/%	M.p./°C
**6a**	2-Py	Me	9.0	74	108–110
**6b**	2-Py	Et	8.0	81	50–51
**6c**	2-Py	Ph	5.0	92	126–128
**6d**	3-Py	Me	10.0	69	116–118
**6e**	3-Py	Et	8.5	78	54–56
**6f**	3-Py	Ph	6.0	88	169–171
**6g**	4-Py	Me	9.0	76	118–120
**6h**	4-Py	Et	8.0	84	62–65
**6i**	4-Py	Ph	4.5	94	172–174

^a^Yield with respect to the starting hydrazide **5a**–**5c**

Our previous studies on the reactions of 3-(2-furyl)acrylohydrazide or 3-(2-thienyl)acrylohydrazide with triethyl orthoesters [45, 57] have shown that the reaction times were relatively shorter (1.5–4 h), what testifies to the higher reactivity of hydrazide reagents containing a furan or thiophene ring in comparison to their pyridine-containing counterparts.

The structures of new products were confirmed with elemental analysis and spectroscopic methods (^1^H and ^13^C NMR, MS, UV, IR). In the series of 2-[2-(pyridyl)ethenyl]-1,3,4-oxadiazoles **6a**–**6i**, the diagnostic signals in the ^1^H NMR spectra are two doublets with the coupling constants *J* = 16.4 Hz associated with two protons of the ethylene group. The value of the coupling constants suggests that *E* geometric isomers of these compounds are formed in the reaction. The proton adjacent to pyridine ring at the β position to the 1,3,4-oxadiazole ring is seen in the range between 7.44 and 7.72 ppm, while the proton α-CH = appears at high fields in the range of 7.08–7.57 ppm. Interestingly, analysing the spectra of 2-[2-(2-pyridyl)ethenyl]-1,3,4-oxadiazoles **6a**–**6c**, one should notice the characteristic ethylene α-CH= and β-CH= proton shifts. These two protons are observed at a much lower field due to the neighbouring pyridine nitrogen atom. Furthermore, the two protons C2″-H and C6″-H of the phenyl group substituted at 5-position of the 1,3,4-oxadiazole ring of **6c**, **6f**, and **6i** are shifted in the ^1^H NMR spectra to lower fields and appear as a doublet of doublets in the range from 8.10 to 8.13 ppm. Such significant changes in the chemical shifts could result from the proximity of these atoms to the ring’s nitrogen and oxygen atoms. In the ^13^C NMR spectra of 1,3,4-oxadiazoles **6a**–**6i**, the characteristic signals are peaks of ethylene carbon atoms α-CH= and β-CH=, which are observed in ranges of 112–114 ppm and 133–139 ppm, respectively. The ring carbon atom C2 is seen in the range between 163 and 166 ppm, while the location of the second carbon atom C5 depends on the type of the substituent and appears between 164 and 170 ppm.

**Fig. 1:**
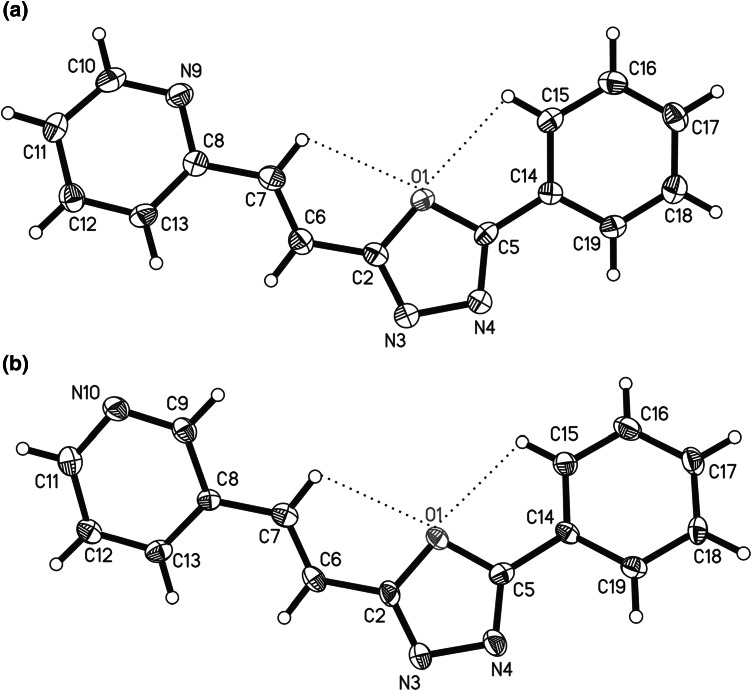
The molecular structure of **a** 5-phenyl-2-[2-(2-pyridyl)ethenyl]-1,3,4-oxadiazole (**6c**), **b** 5-phenyl-2-[2-(3-pyridyl)ethenyl]-1,3,4-oxadiazole (**6f**) showing 50 % displacement ellipsoids (arbitrary spheres for the H atoms). *Dashed lines* indicate intramolecular hydrogen bond

To establish the structure of derivatives **6**, X-ray analysis was also performed. The molecular structure of 5-phenyl-2-[2-(2-pyridyl)ethenyl]-1,3,4-oxadiazole (**6c**) and 5-phenyl-2-[2-(3-pyridyl)ethenyl]-1,3,4-oxadiazole (**6f**) with the atomic numbering scheme is shown in Fig. 1.

Each of the analysed compounds consists of three rings: I (the pyridine ring containing atoms C8–C13), II (the oxadiazole ring containing atoms O1–C5), and III (the phenyl ring containing atoms C14–C19). The values of the I/II, I/III, and II/III dihedral angles are collected in Table 2. According to collected data, it was concluded that both molecules adopt coplanar conformation. The near planarity of the systems favours the formation of intramolecular hydrogen bonds and π-electron delocalization. The C–C bonds located between the aromatic rings (C2–C6, C6–C7, C7–C8, and C5–C14) exhibit intermediate values due to *π*-electron delocalization in the molecules. This effect is more pronounced in the more coplanar structure **6c**.

**Table 2:** Selected geometric parameters for 5-phenyl-2-[2-(2-pyridyl)ethenyl]-1,3,4-oxadiazole (**6c**) and 5-phenyl-2-[2-(3-pyridyl)ethenyl]-1,3,4-oxadiazole (**6f**)

Parameter	**6c**	**6f**
Bond lengths/Å		
C2–C6	1.430 (2)	1.443 (3)
C6–C7	1.329 (2)	1.326 (3)
C7–C8	1.463 (2)	1.459 (3)
C5–C14	1.454 (2)	1.463 (4)
Torsion angles/°		
N3–C2–C6–C7	−177.4 (2)	173.5 (3)
C6–C7–C8–C13	3.6 (2)	−5.4 (4)
N4–C5–C14–C19	8.6 (2)	−15.3 (4)
Dihedral angles/°		
I/II	6.6 (1)	12.1 (2)
I/III	14.7 (8)	27.5 (1)
II/III	8.6 (1)	16.0 (2)

The twist along the C2–C6, C7–C8, and C5–C14 bonds is illustrated by torsion angles and it is rather small in both compounds (Table 2). In the studied molecules, the remaining bond lengths and angles are normal and are in good agreement with the geometry of similar derivatives of 1,3,4-oxadiazole [58–60]. These structures are stabilized by two intramolecular hydrogen bonds C7–H7···O1 and C15–H15A···O1 (Table 3) which give rise to the five-membered ring systems in all cases and confirm existence of *E* geometrical form of both compounds.

**Table 3:** Intramolecular hydrogen bonds geometry for 5-phenyl-2-[2-(2-pyridyl)ethenyl]-1,3,4-oxadiazole (**6c**) and 5-phenyl-2-[2-(3-pyridyl)ethenyl]-1,3,4-oxadiazole (**6f**)

Structure	D–H···A	D_D–H_/Å	D_H···A_/Å	D_D···A_/Å	<(D–H···A)/°
**6c**	C(7)–H(7A)···O(1)	0.93	2.57	2.897 (2)	101.4
**6f**		0.93	2.57	2.903 (3)	101.3
**6c**	C(15)–H(15A)···O(1)	0.93	2.53	2.846 (2)	100.0
**6f**		0.93	2.55	2.860 (3)	99.7

Considering the fact that physical properties of compounds are also strongly dependent on their ability to acid–base interactions, the p*K*_A_ values of 5-substituted 2-[2-(pyridyl)ethenyl]-1,3,4-oxadiazoles **6a**–**6i** were determined (Table 4). The determination of the p*K*_A_ dissociation constants was performed according to the spectrophotometric method of Albert and Serjeant [61] in 50 % aqueous methanol solution (10^−5^ M, room temperature) due to the low solubility of the examined compounds in water. The p*K*_A_ values determined in aqueous methanol solution are lower about 0.6 p*K*_A_ unit comparing with those determined in aqueous solution which is the result of different ionic products of these solvents. Absorption maxima of the 1,3,4-oxadiazole ions were selected as analytical wavelengths bearing in mind their considerable shifts relative to the maxima of the non-protonated forms. The conducted studies have shown that the 1,3,4-oxadiazol-2-ylethenyl moiety at positions 2, 3, or 4 of the pyridine ring causes an increase in acidity compared to the p*K*_A_ value of the unsubstituted pyridine (**7**, Scheme 4) [62].

**Table 4:** p*K*_*A*_ Ionization constants of 2-[2-(pyridyl)ethenyl]-1,3,4-oxadiazoles **6a**–**6i** in aqueous methanol solutions

Compound	Py	*R*	*λ*_ANAL_/nm	p*K*_A_^a^
**6a**	2-Py	Me	300.5	4.03 ± 0.19
**6b**	2-Py	Et	301.0	4.06 ± 0.22
**6c**	2-Py	Ph	312.0	4.10 ± 0.23
**6d**	3-Py	Me	294.0	3.92 ± 0.08
**6e**	3-Py	Et	294.5	3.94 ± 0.16
**6f**	3-Py	Ph	309.5	3.72 ± 0.19
**6g**	4-Py	Me	290.5	4.02 ± 0.15
**6h**	4-Py	Et	291.0	4.09 ± 0.17
**6i**	4-Py	Ph	306.0	4.21 ± 0.12

^a^Determined by spectrophotometric method (H_2_O/MeOH, 1:1 v/v); rt

**Figure:**
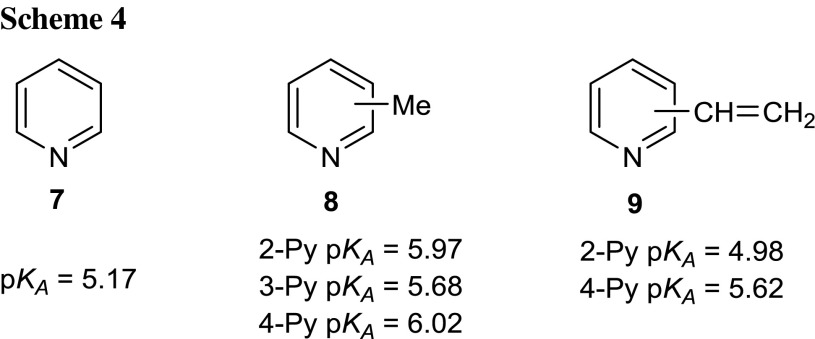

Generally, 2-[2-(pyridyl)ethenyl]-1,3,4-oxadiazoles **6a**–**6i** are stronger acids than the corresponding vinylpyridine derivatives **9** (Scheme 4). This is probably the result of the presence of the electron-withdrawing 1,3,4-oxadiazole ring conjugated via an ethenyl linker to pyridine. This leads to the decreasing of the electron density of the ring nitrogen atom. Among the three series of pyridine substituted 1,3,4-oxadiazoles, 2-[2-(3-pyridyl)ethenyl]-1,3,4-oxadiazoles **6d**–**6f** exhibit the most acidic properties, while the rest **6a**–**6c** and **6g**–**6i** show the similar acid–base activities. Analogical behaviour was observed in the case of differently substituted methylpyridine derivatives **8** (Scheme 4).

## Conclusion

In conclusion, we have synthesized a series of novel 5-substituted 2-[2-(pyridyl)ethenyl]-1,3,4-oxadiazoles in the reactions of three types of differently substituted 3-(pyridyl)acrylohydrazides with triethyl orthoesters in glacial acetic acid. This easy and efficient method has the advantage of providing the desired products in high yields, which makes it a useful addition to the existing synthetic protocols. The presence of the acid-sensitive pyridyl fragment is particularly important because it allows the electronic properties modification of the indicated structures by acid–base interactions, which makes them especially attractive for optoelectronic applications.

## Experimental

All solvents and reagents were purchased from commercial sources and were used without additional purification. Melting points were measured using a Stuart SMP3 melting point apparatus. Elemental analyses were performed with a VarioEL analyser. UV spectra were recorded on a Jasco V-650 spectrophotometer. The ^1^H and ^13^C NMR spectra were recorded on an Agilent 400-MR spectrometer in DMSO-*d*_*6*_, CDCl_3_, or CD_3_OD solutions using TMS as the internal standard. Thin-layer chromatography was performed on silica gel 60 F_254_ (Merck) thin-layer chromatography plates using MeOH/CHCl_3_ (1:4 v/v) as the mobile phase. FT-IR spectra were recorded between 4000 and 650 cm^−1^ on an FT-IR Nicolet 6700 apparatus with a Smart iTR accessory. Mass spectra were obtained on a GC/MS Agilent Technologies 7890A/5975C System with triple axis detector using the EI technique (70 eV). The p*K*_A_ ionization constants of 2-[2-(pyridyl)ethenyl]-1,3,4-oxadiazoles **6a**–**6i** were determined by spectrophotometric method of Albert and Serjeant in 50 % aqueous methanol solutions (10^−5^ M) at room temperature.

### General procedure for the synthesis of 3-(pyridyl)acrylic acids **2a**–**2c**

A mixture of 32.1 g pyridinecarboxaldehyde **1a**–**1c** (0.30 mol) and 31.2 g malonic acid (0.30 mol) in a solution of 30 cm^3^ pyridine and 1 cm^3^ piperidine was heated in a steam bath and stirred for 2 h. After cooling to room temperature, the precipitate was collected by filtration, washed with H_2_O and dried. The crude product was crystallized from a EtOH/H_2_O mixture, yielding the corresponding pure 3-(pyridyl)acrylic acid **2a**–**2c**.

#### *3*-*(2*-*Pyridyl)acrylic acid* (**2a**)

White solid; yield 42.0 g (94 %); m.p.: 201–203 °C (Ref. [63] 202–204 °C).

#### *3*-*(3*-*Pyridyl)acrylic acid* (**2b**)

White solid; yield 41.1 g (92 %); m.p.: 232–234 °C (Ref. [64] 232–235 °C).

#### *3*-*(4*-*Pyridyl)acrylic acid* (**2c**)

White solid; yield 42.9 g (96 %); m.p.: 277–279 °C (Ref. [65] 277–280 °C).

### General procedure for the preparation of 3-(pyridyl)acrylohydrazides **5a**–**5c**

The appropriate 3-(pyridyl)acrylic acid **2a**–**2c** (14.9 g, 0.10 mol) was slowly added to a stirred solution of 5.6 g KOH (0.10 mol) in 100 cm^3^ H_2_O. The mixture was stirred for approximately 10 min and then concentrated using a rotary evaporator. The precipitate was washed with 2 × 50 cm^3^ Et_2_O, collected by filtration and air dried to give the corresponding crude potassium salt as a white solid: 18.1 g (97 %) **3a**, 17.8 g (95 %) **3b**, and 18.3 g (98 %) **3c**.

To a stirred suspension of 16.8 g potassium salt **3a**–**3c** (0.09 mol) in 100 cm^3^ MeCN was added a 1 % solution of pyridine in 30 cm^3^ MeCN and 9.8 g ethyl chloroformate (0.09 mol). The reaction mixture was agitated at 0 °C for 2 h and then slowly poured into a stirred, ice-cooled suspension of 9.0 g hydrazine hydrate (0.18 mol) in 100 cm^3^ MeCN. After filtration, the filtrate was kept in an ice box overnight, then washed with 2 × 50 cm^3^ saturated aqueous Na_2_CO_3_ solution, dried over MgSO_4_, and concentrated using a rotatory evaporator. The crude product was purified by column chromatography with silica gel and an eluent of MeOH/CHCl_3_ (1:4 v/v) to yield the 3-(pyridyl)acrylic acid hydrazides **5a**–**5c**.

#### *3*-*(2*-*Pyridyl)acrylohydrazide* (**5a**, C_8_H_9_N_3_O)

Yellow solid; yield 11.0 g (75 %); m.p.: 108–110 °C; *R*_f_ = 0.31 (MeOH/CHCl_3_, 1:4 v/v); ^1^H NMR (400 MHz, DMSO-*d*_6_): *δ* = 9.53 (1H, s, NH), 8.58 (1H, d, *J* = 4.0 Hz, C6′-H), 7.80 (1H, t, *J* = 7.6 Hz, C4′-H), 7.54 (1H, d, *J* = 7.6 Hz, C3′-H), 7.45 (1H, d, *J* = 15.6 Hz, β-CH=), 7.34–7.31 (1H, m, C5′-H), 7.00 (1H, d, *J* = 15.6 Hz, α-CH=), 4.51 (2H, br s, NH_2_) ppm; ^13^C NMR (100.6 MHz, DMSO-*d*_6_): *δ* = 164.0 (CO), 153.0, 149.8, 137.6, 137.2, 124.2, 124.0, 123.9 ppm; IR (ATR): $\bar{V}$ = 3,277, 3,176, 3,054, 3,026, 1,989, 1,659, 1,591, 1,486, 1,474, 1,437, 1,336, 1,305, 1,249, 1,218, 1,188, 1,126, 1,095, 1,048, 955, 922, 892, 872, 857, 728, 676 cm^−1^; UV–Vis (MeOH): *λ*_max_ (*ε* × 10^−3^) = 291.0 (13.65), 250.0 (13.46), 201.0 (16.65) nm (mol^−1^ dm^3^ cm^−1^); MS (EI, 70 eV): *m*/*z* (%) = 163 (M^+^, 18), 148 (22), 133 (79), 132 (100), 130 (27), 105 (21), 104 (92), 79 (40), 78 (65), 77 (13), 76 (10), 52 (15), 51 (30).

#### *3*-*(3*-*Pyridyl)acrylohydrazide* (**5b**)

Yellow solid; yield 10.7 g (73 %); m.p.: 128–130 °C (Ref. [66] 126 °C).

#### *3*-*(4*-*Pyridyl)acrylohydrazide* (**5c**)

Yellow solid; yield 11.6 g (79 %); m.p.: 149–151 °C (Ref. [67] 150–151 °C).

### General procedure for the synthesis of 5-substituted 2-[2-(pyridyl)ethenyl]-1,3,4-oxadiazoles **6a**–**6i**

The starting 3-(pyridyl)acrylohydrazide **5a**–**5c** (1.63 g, 10.0 mmol) was added to a mixture of the appropriate triethyl orthoester (20.0 mmol) and 10 cm^3^ glacial AcOH. The mixture was kept under reflux until the starting hydrazide was fully consumed (monitored by TLC, 4.5–10 h). After cooling, the excessive orthoester and AcOH were evaporated under reduced pressure. The crude products **6a**–**6i** were crystallized from appropriate solvents.

#### *5*-*Methyl*-*2*-*[2*-*(2*-*pyridyl)ethenyl]*-*1,3,4*-*oxadiazole* (**6a**, C_10_H_9_N_3_O)

White solid; yield 1.38 g (74 %); m.p.: 108–110 °C; *R*_f_ = 0.66 (MeOH/CHCl_3_, 1:4 v/v); ^1^H NMR (400 MHz, CD_3_OD): *δ* = 8.66 (1H, d, *J* = 4.0 Hz, C6′-H), 7.91 (1H, t, *J* = 7.6 Hz, C4′-H), 7.71 (1H, d, *J* = 7.6 Hz, C3′-H), 7.66 (1H, d, *J* = 16.4 Hz, β-CH=), 7.55 (1H, d, *J* = 16.4 Hz, α-CH=), 7.45–7.42 (1H, m, C5′-H), 2.65 (3H, s, CH_3_) ppm; ^13^C NMR (100.6 MHz, CD_3_OD): *δ* = 165.8, 165.7, 154.1, 150.9, 138.9, 138.8, 125.6, 125.2, 114.5, 10.7 (CH_3_) ppm; IR (ATR): $\bar{V}$ = 3,038, 2,162, 1,648, 1,577, 1,529, 1,476, 1,437, 1,390, 1,353, 1,324, 1,230, 1,162, 1,100, 1,041, 991, 986, 904, 792, 675, 665 cm^−1^; UV–Vis (MeOH): *λ*_max_ (*ε* × 10^−3^) = 302.0 (21.17), 263.5 (14.13), 201.5 (9.96) nm (mol^−1^ dm^3^ cm^−1^); MS (EI, 70 eV): *m*/*z* (%) = 187 (M^+^, 40), 186 (100), 144 (12), 132 (15), 117 (17), 116 (34), 104 (12), 90 (11), 78 (14), 51 (10).

#### *5*-*Ethyl*-*2*-*[2*-*(2*-*pyridyl)ethenyl]*-*1,3,4*-*oxadiazole* (**6b**, C_11_H_11_N_3_O)

White solid; yield 1.63 g (81 %); m.p.: 50–51 °C; *R*_f_ = 0.68 (MeOH/CHCl_3_, 1:4 v/v); ^1^H NMR (400 MHz, CD_3_OD): *δ* = 8.66 (1H, d, *J* = 4.0 Hz, C6′-H), 7.91 (1H, t, *J* = 7.6 Hz, C4′-H), 7.71 (1H, d, *J* = 7.6 Hz, C3′-H), 7.66 (1H, d, *J* = 16.4 Hz, β-CH=), 7.56 (1H, d, *J* = 16.4 Hz, α-CH=), 7.45–7.42 (1H, m, C5′-H), 3.01 (2H, q, *J* = 7.6 Hz, CH_2_), 1.46 (3H, t, *J* = 7.6 Hz, CH_3_) ppm; ^13^C NMR (100.6 MHz, CD_3_OD): *δ* = 169.7, 165.5, 154.1, 150.9, 138.9, 138.8, 125.6, 125.2, 114.5, 19.8 (CH_2_), 10.8 (CH_3_) ppm; IR (ATR): $\bar{V}$ = 3,188, 3,062, 1,674, 1,596, 1,554, 1,502, 1,471, 1,420, 1,355, 1,322, 1,288, 1,230, 1,178, 1,134, 1,099, 1,070, 1,037, 967, 919, 891, 841, 829, 673, 662 cm^−1^; UV–Vis (MeOH): *λ*_max_ (*ε* × 10^−3^) = 302.5 (17.44), 264.5 (11.03), 200.5 (7.53) nm (mol^−1^ dm^3^ cm^−1^); MS (EI, 70 eV): *m*/*z* (%) = 201 (M^+^, 46), 200 (13), 144 (32), 132 (22), 117 (28), 116 (100), 104 (13), 90 (10), 89 (14), 78 (21), 57 (17).

#### *5*-*Phenyl*-*2*-*[2*-*(2*-*pyridyl)ethenyl]*-*1,3,4*-*oxadiazole* (**6c**, C_15_H_11_N_3_O)

White solid; yield 2.29 g (92 %); m.p.: 126–128 °C; *R*_f_ = 0.70 (MeOH/CHCl_3_, 1:4 v/v); ^1^H NMR (400 MHz, CD_3_OD): *δ* = 8.61 (1H, d, *J* = 4.0 Hz, C6′-H), 8.10 (2H, dd, *J* = 8.0, 1.6 Hz, C2″-H, C6″-H), 7.86 (1H, t, *J* = 7.6 Hz, C4′-H), 7.72 (1H, d, *J* = 16.4 Hz, β-CH=), 7.67 (1H, d, *J* = 7.6 Hz, C3′-H), 7.61-7.58 (3H, m, C3″-H, C4″-H, C5″-H), 7.57 (1H, d, *J* = 16.4 Hz, α-CH=), 7.39–7.36 (1H, m, C5′-H) ppm; ^13^C NMR (100.6 MHz, CD_3_OD): *δ* = 165.7, 165.3, 153.9, 150.8, 139.2, 138.6, 133.3, 130.3, 127.9, 125.5, 125.2, 124.4, 114.3 ppm; IR (ATR): $\bar{V}$ = 2,919, 2,850, 1,969, 1,667, 1,585, 1,567, 1,519, 1,473, 1,450, 1,430, 1,373, 1,315, 1,275, 1,221, 1,190, 1,152, 1,093, 1,071, 1,015, 993, 973, 845, 830, 744, 690 cm^−1^; UV–Vis (MeOH): *λ*_max_ (*ε* × 10^−3^) = 312.0 (17.73), 245.5 (8.18), 201.5 (16.65) nm (mol^−1^ dm^3^ cm^−1^); MS (EI, 70 eV): *m*/*z* (%) = 249 (M^+^, 55), 193 (14), 192 (17), 144 (50), 132 (26), 116 (83), 105 (100), 104 (17), 90 (12), 78 (28), 77 (70), 63 (12), 51 (20).

#### *5*-*Methyl*-*2*-*[2*-*(3*-*pyridyl)ethenyl]*-*1,3,4*-*oxadiazole* (**6d**, C_10_H_9_N_3_O)

White solid; yield 1.29 g (69 %); m.p.: 116–118 °C; *R*_f_ = 0.53 (MeOH/CHCl_3_, 1:4 v/v); ^1^H NMR (400 MHz, CDCl_3_): *δ* = 8.76 (1H, d, *J* = 2.0 Hz, C2′-H), 8.61 (1H, dd, *J* = 4.8, 1.6 Hz, C6′-H), 7.89 (1H, dt, *J* = 8.0, 2.0 Hz, C4′-H), 7.50 (1H, d, *J* = 16.4 Hz, β-CH=), 7.37 (1H, dd, *J* = 8.0, 4.8 Hz, C5′-H), 7.08 (1H, d, *J* = 16.4 Hz, α-CH=), 2.60 (3H, s, CH_3_) ppm; ^13^C NMR (100.6 MHz, CDCl_3_): *δ* = 163.9, 163.4, 150.6, 149.2, 134.7, 133.4, 130.5, 123.8, 112.1, 11.0 (CH_3_) ppm; IR (ATR): $\bar{V}$ = 3,050, 3,019, 2,164, 1,645, 1,575, 1,524, 1,503, 1,484, 1,446, 1,419, 1,362, 1,236, 1,131, 1,051, 1,024, 967, 954, 859, 819, 800, 747, 717, 674, 664 cm^−1^; UV–Vis (MeOH): *λ*_max_ (*ε* × 10^−3^) = 293.5 (21.38), 283.5 (21.64), 200.5 (10.05) nm (mol^−1^ dm^3^ cm^−1^); MS (EI, 70 eV): *m*/*z* (%) = 187 (M^+^, 34), 186 (100), 144 (10), 132 (12), 116 (28), 104 (11), 90 (12), 51 (10), 43 (22).

#### *5*-*Ethyl*-*2*-*[2*-*(3*-*pyridyl)ethenyl]*-*1,3,4*-*oxadiazole* (**6e**, C_11_H_11_N_3_O)

White solid; yield 1.57 g (78 %); m.p.: 54–56 °C; *R*_f_ = 0.61 (MeOH/CHCl_3_, 1:4 v/v); ^1^H NMR (400 MHz, CDCl_3_): *δ* = 8.77 (1H, d, *J* = 2.0 Hz, C2′-H), 8.61 (1H, dd, *J* = 4.8, 1.6 Hz, C6′-H), 7.90 (1H, dt, *J* = 8.0, 2.0 Hz, C4′-H), 7.50 (1H, d, *J* = 16.4 Hz, β-CH=), 7.38 (1H, dd, *J* = 8.0, 4.8 Hz, C5′-H), 7.10 (1H, d, *J* = 16.4 Hz, α-CH=), 2.94 (2H, q, *J* = 7.6 Hz, CH_2_), 1.44 (3H, t, *J* = 7.6 Hz, CH_3_) ppm; ^13^C NMR (100.6 MHz, CDCl_3_): *δ* = 167.5, 163.7, 150.6, 149.3, 134.6, 133.3, 130.6, 123.8, 112.2, 19.1 (CH_2_), 10.7 (CH_3_) ppm; IR (ATR): $\bar{V}$ = 3,178, 3,073, 1,645, 1,570, 1,504, 1,463, 1,450, 1,412, 1,379, 1,344, 1,315, 1,248, 1,193, 1,129, 1,083, 1,024, 955, 860, 806, 792, 699, 675 cm^−1^; UV–Vis (MeOH): *λ*_max_ (*ε* × 10^−3^) = 294.5 (22.24), 284.0 (22.31), 201.0 (11.39) nm (mol^−1^ dm^3^ cm^−1^); MS (EI, 70 eV): *m*/*z* (%) = (M^+^, 32), 200 (100), 144 (10), 132 (12), 116 (28), 104 (11), 57 (14).

#### *5*-*Phenyl*-*2*-*[2*-*(3*-*pyridyl)ethenyl]*-*1,3,4*-*oxadiazole* (**6f**)

White solid; yield 2.19 g (88 %); m.p.: 169–171 °C (Ref. [68] 170–172 °C).

#### *5*-*Methyl*-*2*-*[2*-*(4*-*pyridyl)ethenyl]*-*1,3,4*-*oxadiazole* (**6g**, C_10_H_9_N_3_O)

White solid; yield 1.42 g (76 %); m.p.: 118–120 °C; *R*_f_ = 0.55 (MeOH/CHCl_3_, 1:4 v/v); ^1^H NMR (400 MHz, CDCl_3_): *δ* = 8.67 (2H, d, *J* = 6.0 Hz, C2′-H, C6′-H), 7.44 (1H, d, *J* = 16.4 Hz, β-CH=), 7.40 (2H, d, *J* = 6.0 Hz, C3′-H, C5′-H), 7.20 (1H, d, *J* = 16.4 Hz, α-CH=), 2.61 (3H, s, CH_3_) ppm; ^13^C NMR (100.6 MHz, CDCl_3_): *δ* = 163.7, 163.6, 150.5, 141.8, 135.6, 121.2, 114.3, 11.0 (CH_3_) ppm; IR (ATR): $\bar{V}$ = 3,188, 3,061, 2,168, 1,946, 1,674, 1,646, 1,577, 1,557, 1,507, 1,471, 1,439, 1,402, 1,360, 1,323, 1,287, 1,233, 1,220, 1,196, 1,134, 1,097, 1,049, 962, 914, 890, 880, 746, 729, 709, 674, 663 cm^−1^; UV–Vis (MeOH): *λ*_max_ (*ε* × 10^−3^) = 291.0 (23.16), 282.0 (24.45), 209.5 (9.42) nm (mol^−1^ dm^3^ cm^−1^); MS (EI, 70 eV): *m*/*z* (%) = 187 (M^+^, 66), 186 (100), 132 (17), 130 (12), 117 (10), 104 (11), 90 (16), 89 (10), 78 (11), 51 (13).

#### *5*-*Ethyl*-*2*-*[2*-*(4*-*pyridyl)ethenyl]*-*1,3,4*-*oxadiazole* (**6h**, C_11_H_11_N_3_O)

White solid; yield 1.69 g (84 %); m.p.: 62–65 °C; *R*_f_ = 0.59 (MeOH/CHCl_3_, 1:4 v/v); ^1^H NMR (400 MHz, CDCl_3_): *δ* = 8.68 (2H, d, *J* = 6.0 Hz, C2′-H, C6′-H), 7.44 (1H, d, *J* = 16.4 Hz, β-CH=), 7.40 (2H, d, *J* = 6.0 Hz, C3′-H, C5′-H), 7.22 (1H, d, *J* = 16.4 Hz, α-CH=), 2.95 (2H, q, *J* = 7.6 Hz, CH_2_), 1.44 (3H, t, *J* = 7.6 Hz, CH_3_) ppm; ^13^C NMR (100.6 MHz, CDCl_3_): *δ* = 167.3, 163.9, 150.5, 141.7, 135.6, 121.2, 114.3, 19.2 (CH_2_), 10.8 (CH_3_) ppm; IR (ATR): $\bar{V}$ = 3,194, 3,082, 1,646, 1,587, 1,549, 1,502, 1,484, 1,411, 1,335, 1,291, 1,227, 1,170, 1,124, 1,092, 1,060, 1,037, 967, 925, 871, 821, 677, 662 cm^−1^; UV–Vis (MeOH): *λ*_max_ (*ε* × 10^−3^) = 292.0 (24.28), 282.5 (25.75), 210.0 (10.18) nm (mol^−1^ dm^3^ cm^−1^); MS (EI, 70 eV): *m*/*z* (%) = 201 (M^+^, 52), 200 (100), 132 (16), 116 (23), 104 (11), 90 (14), 89 (10), 78 (21), 57 (17).

#### *5*-*Phenyl*-*2*-*[2*-*(4*-*pyridyl)ethenyl]*-*1,3,4*-*oxadiazole* (**6i**, C_15_H_11_N_3_O)

White solid; yield 2.34 g (94 %); m.p.: 172–174 °C; *R*_f_ = 0.67 (MeOH/CHCl_3_, 1:4 v/v); ^1^H NMR (400 MHz, CDCl_3_): *δ* = 8.69 (2H, d, *J* = 6.0 Hz, C2′-H, C6′-H), 8.13 (2H, dd, *J* = 8.0, 1.6 Hz, C2″-H, C6″-H), 7.56 (1H, d, *J* = 16.4 Hz, β-CH=), 7.55–7.51 (3H, m, C3″-H, C4″-H, C5″-H), 7.43 (2H, d, *J* = 6.0 Hz, C3′-H, C5′-H), 7.29 (1H, d, *J* = 16.4 Hz, α-CH=) ppm; ^13^C NMR (100.6 MHz, CDCl_3_): *δ* = 164.5, 163.3, 150.6, 141.8, 135.9, 132.0, 129.1, 127.0, 123.4, 121.2, 114.3 ppm; IR (ATR): $\bar{V}$ = 3,188, 3,062, 1,962, 1,674, 1,596, 1,555, 1,502, 1,471, 1,420, 1,356, 1,322, 1,288, 1,230, 1,178, 1,134, 1,099, 1,070, 1,038, 971, 918, 891, 880, 817, 728, 663 cm^−1^; UV–Vis (MeOH): *λ*_max_ (*ε* × 10^−3^) = 307.0 (29.03), 248.0 (13.56), 202.0 (26.35) nm (mol^−1^ dm^3^ cm^−1^); MS (EI, 70 eV): *m*/*z* (%) = 249 (M^+^, 57), 248 (100), 132 (14), 105 (47), 78 (10), 77 (43), 51 (13).

### X-ray crystal structure analysis for **6c** and **6f**

The single crystal of 5-phenyl-2-[2-(2-pyridyl)ethenyl]-1,3,4-oxadiazole (**6c**) and 5-phenyl-2-[2-(3-pyridyl)ethenyl]-1,3,4-oxadiazole (**6f**) were used for data collection at 100.0(1) K on a four-circle Oxford Diffraction Xcalibur diffractometer equipped with a two-dimensional area CCD detector using graphite-monochromatized MoK_α_ radiation (*λ* = 0.71073 Å) and the *ω*-scan technique. Integration of the intensities and correction for Lorenz and polarization effects were performed using CrysAlis RED software [69]. The crystal structures were solved by direct methods and refined by a full-matrix least-squares method on *F* ^2^ using the program SHELXL-97 [70].

Complete crystallographic details for **6c** and **6f** are available as Supplementary data (CCDC 994,076 and 994,077) and have been deposited at the Cambridge Crystallographic Data Centre (CCDC), 12 Union Road, Cambridge, CB21EZ, UK; e-mail: deposit@ccdc.cam.ac.uk or http://www.ccdc.cam.ac.uk. Any request to the CCDC for this material should quote the full literature citation.
